# Low Velocity Impact Localization of Variable Thickness Composite Laminates

**DOI:** 10.3390/s21186103

**Published:** 2021-09-12

**Authors:** Guan Lu, Yuchen Zhou, Yiming Xu

**Affiliations:** 1 School of Mechanical Engineering, Nantong University, Nantong 226000, China; luguan@ntu.edu.cn (G.L.); 2009310024@stmail.ntu.edu.cn (Y.Z.); 2 School of Electrical Engineering, Nantong University, Nantong 226000, China

**Keywords:** fiber Bragg grating, variable thickness composite laminates, impact localization, empirical mode decomposition, zero-mean normalized cross-correlation, thickness correction

## Abstract

Variable thickness composite laminates (VTCL) are susceptible to impact during use and may result in irreparable internal damage. In order to locate the internal impact damage of complex composite structures and monitor the impact signals of VTCL at the same time, a low velocity impact (LVI) monitoring system based on an optical fiber sensing network was constructed. Fiber Bragg grating (FBG) sensors are suitable for monitoring strain characteristics. By arranging FBG sensors on the laminate, we studied the spectrum analysis and localization of the impact signal collected by a FBG demodulator at constant temperature. The prior knowledge of variable thickness composite structures is difficult to obtain, and the multi-sensor dynamic monitoring is complex and difficult to realize. In order to locate the LVI of composite structures without prior knowledge, based on empirical mode decomposition (EMD), we proposed an impact localization method with zero-mean normalized cross-correlation (ZNCC) and thickness correction. The experimental results of LVI localization verification show that the ZNCC algorithm can effectively remove the temperature cross-sensitivity and impact energy influencing factors, and the thickness correction can reduce the interference of variable thickness characteristics on localization performance. The maximum localization error is 24.41 mm and the average error is 15.67 mm, which meets engineering application requirements. The method of variable-thickness normalization significantly improves impact localization performance for VTCL.

## 1. Introduction

In recent years, composite materials have been widely used in aviation, machinery, medicine, and other fields due to their excellent mechanical properties such as high strength and high ratio. The consequent problem is the need for dynamic monitoring and maintenance of composite materials in service [1,2,3]. Because composite materials are often used as laminated plates, the LVI to the structure may cause the internal delamination and fracture, and these internal damages are not easy to detect. The damage hidden in the composite structure will lead to a great degradation of the mechanical properties, and even lead to a catastrophic failure of the entire structure [4,5]. Therefore, it is very important to monitor the dynamic signals of composite structures.

Nevertheless, the daily inspection and maintenance of composite structures will consume a lot of time and cost. Therefore, real-time impact signal monitoring of complex composite structures can provide an early warning of structural damage and greatly reduce cost investment [6]. In order to detect various forms of internal damage of composite structures, researchers have adopted a variety of non-destructive testing (NDT) methods, including eddy current, ultrasound, radiation, thermal imaging, Lamb wave, and so on [7,8]. However, the NDT methods usually need to suspend the overall structure operation, which greatly reduces the operability for large structures. Therefore, real-time monitoring the external impact of the structure can effectively assess the impact location and reduce the cost of NDT [9]. Researchers have used a variety of advanced sensing technology and signal processing algorithms to study impact localization, most of which require prior knowledge, such as the geometry or group velocity of the target structure [10,11].

In order to overcome these shortcomings of the traditional impact localization methods, many studies have adopted intelligent algorithms such as neural networks [12]. These methods can locate external impact of various structures without prior knowledge. Jeong et al. proposed a composite plate impact localization technology based on acoustic emission by using a time reversal method [13]. Park et al. used piezoelectric sensors and laser Doppler vibrometers to locate external impact on complex structures also by the time reversal method [14]. Ciampa et al. proposed an in situ structure imaging method, which can detect the real-time impact position of complex composite structures [15]. These studies have successfully identified impact locations by using some methods without priori knowledge, but rarely involve optical fiber sensing technology.

At present, there are many kinds of sensors used for structural dynamic load monitoring, among which fiber grating sensor has the advantages of small volume, light weight, anti-interference, and corrosion resistance [16]. It is suitable for nondestructive testing and dynamic detection of composite structures [17,18,19]. Jang et al. used a high-speed demodulator to collect impact response signals, and proposed a rapid impact localization algorithm combined with the artificial neural network method [20]. Jang et al. conducted impact localization for composite stiffened panels by comparing the root mean square values between the reference signals and the acquired impact signals [21]. Hiche et al. studied a localization algorithm based on strain amplitude, and located the impact source by comparing the absolute value of the maximum strain amplitude of the impact signal measured by the fiber grating sensor [22]. Webb et al. used an FBG sensor to measure the full spectrum of composite laminates in a dynamic environment, and realized the health monitoring of composite structures by analyzing the spectrum distortion of thin plate in vibration, impact, and damage [23]. Kim et al. devised a localization algorithm used the normalized cross-correlation method to compare the similarities of the two signals, and localized low-speed impact on a stiffened composite structure by using four FBG sensors [24]. Kim et al. proposed an error-outlier-based algorithm with Pugh’s concept selection that can choose the impact point with the lowest error among several candidates by using FBG sensors [25]. Jang et al. used the magnitudes of FBG sensor signals to estimate the distances between each sensor and impact location, and then used the triangulation method to locate the impact sources [26]. Yu et al. proposed an impact localization algorithm based on recurrence quantification analysis and FBG sensors, which effectively predicted the location of impact on composite structure with a low sampling rate interrogation module and small samples [27]. However, the current research is less related to the impact monitoring and localization of variable thickness composite structures.

The strength of VTCL is higher than that of common composite laminates, but signal analysis and impact localization under dynamic loading are more complicated. The research on external impact of VTCL can not only promote the development of damage mechanism and tolerance design method, but also enhance the level of composite structural design. To locate the external impact of VTCL, we proposed a localization method based on EMD, ZNCC, and thickness correction without prior knowledge. The EMD algorithm can effectively extract the useful feature information from the impact signals, while the ZNCC algorithm can effectively remove the temperature cross-sensitivity and impact energy influencing factors of the sensor, and the thickness correction can reduce the interference of variable thickness characteristics on localization performance.

Considering the complexity of impact signal analysis and prior knowledge acquisition for variable thickness composite structures, firstly, we studied the impact signals of VTCL based on FBG sensing technology, EMD algorithm, and signal spectrum analysis. Secondly, we realized the impact localization of VTCL based on ZNCC algorithm and thickness correction. Thirdly, the verification experiment was carried out on the surface of composite wing oil tank. In addition, the feasibility and performance of the impact localization method were also studied.

## 2. Impact Localization Algorithm Based on an FBG Sensing Network

### 2.1. FBG Strain Sensing Principle

Fiber Bragg gratings produce a narrow-band reflection of incident light that satisfies grating reflection conditions. The basic principle is [28]:

(1) $$\lambda =2n_{eff}\Lambda$$

λ is the reflection peak wavelength; Λ is the grating period; and $n_{eff}$ is the effective refractive index.

Due to the stretching of the grating period and the elastic-optic effect, the grating has a wavelength change when it is subjected to stress. Assuming that the sensor is only subjected to tensile strain ϵ at the moment, from the grating period and Equation (1), there is:

(2) $$\frac{\Delta \lambda }{\lambda }=\left(1-P_{e}\right)\cdot \varepsilon$$

$P_{e}$ is an effective elastic-optic function. According to Equation (2), there is a linear relationship between the grating wavelength shift and longitudinal strain at constant temperature.

The effect of impact load on structural stress can cause a center wavelength shift of the FBG sensor. Therefore, the FBG sensor can be used in the impact study of VTCL.

### 2.2. FBG Sensor Impact Signal Analysis Method

The classical Fourier transform method is often used to analyze the spectrum of impact signals detected by grating sensors; however, considering the complexity of the spectrum of VTCL, we decompose the impact signals by the EMD algorithm in the Hilbert–Huang Transform (HHT) for spectrum analysis. The HHT proposed by Norden E. Huang in 1998 is mainly composed of EMD decomposition and Hilbert spectrum. EMD algorithm can directly decompose several intrinsic mode functions (IMFs) from high frequency to low frequency from complex time series, that is, basic time series. In most studies of practical engineering applications of HHT, researchers used EMD for spectrum analysis. The impact response of VCTL is non-stationary and often disturbed by structural vibration and other interference signals. In view of this, the EMD algorithm in HHT can decompose one complex non-stationary signal into the sum of IMF components [29].

#### 2.2.1. Empirical Mode Decomposition

The main function of EMD is to remove the signal superimposed wave and make data waveforms more symmetrical. The original signal M(t) can be decomposed into:

(3) $$M\left(t\right)=\sum_{i=1}^{n}s_{i}\left(t\right)+r_{n}\left(t\right)$$

The termination conditions for the EMD screening process are: $s_{n}\left(t\right)$ or $r_{n}\left(t\right)$ is less than a predetermined error; or $r_{n}\left(t\right)$ is a monotone function. Where $r_{n}\left(t\right)$ indicates the signal trend, $s_{n}\left(t\right)$ is the decomposition component, and the components of each order are approximately orthogonal and adaptive.

#### 2.2.2. Decomposition Procedure

EMD does not need a priori substrate and has adaptability. The frequency components in each IMF are only related to the sampling frequency and the signal itself.

Specific steps of EMD are as follows:

1. Decompose the original signals using EMD, obtain the correlation coefficient of each IMF and the original signal according to the following formula. The solution process is as follows: if there are two sets of data with the same number, they are recorded as $x_{1}$, $x_{2}$, $x_{1}$ is a row vector, $x_{2}$ is a column vector, and the correlation coefficient is:
  (4) $$q=\frac{\sum x_{1}x_{2}-\frac{1}{n}\sum x_{1}\sum x_{2}}{\sqrt{\left[\sum x_{1}^{2}-\frac{1}{n}{\left(\sum x_{1}\right)}^{2}\right]\left[\sum x_{2}^{2}-\frac{1}{n}{\left(\sum x_{2}\right)}^{2}\right]}}$$
2. Select the IMF with larger *q* for analysis, and extract the impact signal feature of composite laminates.

The impact response depends on the damping, stiffness, natural frequency, excitation and other conditions of the structure. We can apply the EMD algorithm to the impact signal decomposition of VTCL, and study the characteristics of first few IMF components, the useful feature information of the impact signal can be effectively extracted after ignoring some components with low amplitude and certain frequency.

### 2.3. Impact Localization Algorithm of Variable Thickness Composite Laminate

The ZNCC algorithm is sensitive to the change of conditions, therefore, it is suitable for the impact localization research of VTCL. The feature component obtained by EMD can be directly used to establish a ZNCC model without further processing. After extracting the feature vectors of the impact signals of the specimen, it is necessary to conduct thickness correction, and we can locate the impact position by using a reference area centroid method.

#### 2.3.1. Zero-Mean Normalized Cross-Correlation Algorithm

The normalized cross-correlation algorithm is a signal processing method, which is used to represent the similarity between two different signals. When the waveforms and phases of the two signals are similar, the cross-correlation value is relatively high. In this paper, there are two steps: remove the mean value of the impact feature vector obtained by EMD and calculate the normalized cross-correlation of the features.

The mean value $\bar{m}$ of the feature vector *m* becomes 0 when the impact feature vector obtained by EMD is mean-removed, and the feature vector after mean removed is $m^{\prime }$. Under the conditions of different temperature, the same position and the same energy, the mean values of the impact feature vectors obtained by the EMD are different, but the signal waveform and time domain amplitude are similar. Therefore, the zero-mean step can be used to remove the cross interference of temperature factors to the center wavelength of the sensor.

The zero-mean cross-correlation between the feature vectors $m_{1}$ and $m_{2}$ is defined as:

(5) $$\left(m_{1}*m_{2}\right)\left(\tau \right)=\int_{-\infty }^{\infty }m_{1}^{\prime }\left(t\right)m_{2}^{\prime }\left(t+\tau \right)dt$$

where ∗ represents the zero-mean cross-correlation operation, *t* is the time parameter of the signal, and τ is the time delay between two signals.

In order to unify the signal benchmark, it is necessary to convert the zero-mean impact feature vector, that is, normalize it. Then, the ZNCC operation between the two feature vectors is expressed as:

(6) $$\left(\frac{m_{1}}{M_{1}}*\frac{m_{2}}{M_{2}}\right)\left(\tau \right)$$

wherein the zero-mean normalization constant $M_{1}$ of the feature vector $m_{1}$ is the square root of the total energy in the time domain of $m_{1}$, expressed as follows:

(7) $$M_{1}=\int_{-\infty }^{\infty }\left|m_{1}^{\prime }\left(t\right)\right|dt$$

Similarly, the zero-mean normalization constant of the feature vector $m_{2}$ can be expressed as:

(8) $$M_{2}=\int_{-\infty }^{\infty }\left|m_{2}^{\prime }\left(t\right)\right|dt$$

If the two impact feature vectors have the same impact locations and the different impact energy, the waveforms of the feature vectors are similar, but the amplitudes are different. Ideally, the relationship between the two impact feature vectors can be expressed as:

(9) $$m_{1}\left(t\right)=n\cdot m_{2}\left(t\right)$$

where *n* is a constant.

Substitute Equation (9) into Equation (6) to obtain the ZNCC operation between the two feature vectors as:

(10) $$\left(\frac{m_{1}}{M_{1}}*\frac{m_{2}}{M_{2}}\right)\left(\tau \right)=\frac{1}{M_{1}\left(nM_{1}\right)}\left(m_{1}^{\prime }*nm_{1}^{\prime }\right)\left(\tau \right)=\frac{1}{M_{1}^{2}}\left(m_{1}^{\prime }*m_{1}^{\prime }\right)\left(\tau \right)$$

when τ is 0, and Equation (10) has a maximum value of 1.

The above derivation process indicates that the signal features of impact location can be effectively extracted by using ZNCC algorithm. Impact signal features can be used to preliminarily evaluate the actual location of the impact load.

#### 2.3.2. Impact Location Algorithm Procedure

In the process of impact localization, the impact location is evaluated by the cross-correlation results between feature vectors of the sample signal and the to-be-identified signal. Firstly, all the impact sample signals and the to-be-identified signals are decomposed by EMD algorithm, and the extracted impact feature vectors are zero-mean normalized. Secondly, due to the influence of thickness factors on the variable thickness composite structure, the integrated thickness coefficient is calculated by the first-order EMD component. The integrated thickness coefficient is used to correct the zero-mean normalized vector and greatly reduce the effect of impact energy on the localization accuracy of VTCL. Thirdly, the comprehensive cross-correlation value between the sample feature vector and the to-be-identified feature vector is calculated, that is, the sum of the cross-correlation values calculated in the sensor network. Finally, since the sample points near the actual impact point will show higher cross-correlation values, the samples with the first four largest cross-correlation values are selected to determine the localization reference area for evaluating the impact position. In summary, the specific steps of impact localization algorithm for VTCL are as follows:

1. Collect data at *C* impact locations, and the impact signal vectors collected by *Q* sensors at each location form a signal matrix $M_{i}=(m_{i1},m_{i2},\cdots ,m_{k},\cdots ,$$m_{iQ}),$ (where *i* = 1, 2, 3, ⋯, *C*) as impact sample signal. The signal vector $m_{k}$ is decomposed by EMD, then calculate the correlation coefficient *q* between the component $s_{j}\left(t\right)$ and the original signal $m_{k}$. Select the component $s_{x}\left(t\right)$ with a larger *q* as the feature component $s_{m_{k}}$ and get the signal feature matrix $u_{i}=\left(s_{m_{i1}},s_{m_{i2}},\cdots ,s_{m_{k}},\cdots ,s_{m_{iQ}}\right)$ (where *i* = 1, 2, 3, ⋯, *C*) in the same way.
2. The impact sample feature vector obtained after mean removal is $u_{i}^{\prime }=(s_{m_{i1}}^{\prime },s_{m_{i2}}^{\prime },$$\cdots ,s_{m_{k}}^{\prime },\cdots ,s_{m_{iQ}}^{\prime })$ (where $s_{m_{ii}}^{\prime }=s_{m_{ii}}-{\bar{s}}_{m_{ii}}$, *i* = 1, 2, 3,⋯, *C*, *j* = 1, 2, 3, ⋯, *Q*), whose zero-mean normalization vector $U_{i}=(S_{m_{i1}},S_{m_{i2}},$$\cdots ,S_{m_{k}},\cdots ,S_{m_{iQ}})$ consists of the following normalization constants:
  (11) $$S_{ij}=\int_{-\infty }^{\infty }\left|s_{ij}^{\prime }\left(t\right)\right|dt$$
3. Perform spectrum analysis on the EMD component $s_{1}\left(t\right)$ of the signal vector $m_{k}$ in 1) to obtain the number $b_{k}$ of the peaks, where the peak definition threshold is $V=f_{k}/a$, and *a* is the number of component vectors. Similarly, the peak number set $B_{i}=\left(b_{i1},b_{i2},\cdots ,b_{k},\cdots ,b_{iQ}\right)$ (where *i* = 1, 2, 3, ⋯, *C*) of the signal matrix $M_{i}$ can be obtained. After normalizing $B_{i}$, the set of integrated thickness coefficients $J_{i}=\left(j_{i1},j_{i2},\cdots ,j_{k},\cdots ,j_{iQ}\right)$ (where *i* = 1, 2, 3, ⋯, *C*) of $M_{i}$ is obtained. Using $J_{i}$ to correct $u_{i}^{\prime }$, we can obtain sample impact feature vector $u_{i}^{\prime \prime }=\left(s_{m_{i1}}^{\prime \prime },s_{m_{i2}}^{\prime \prime },\cdots ,s_{m_{k}}^{\prime \prime },\cdots ,s_{m_{iQ}}^{\prime \prime }\right)$ of the VTCL, where $s_{m_{k}}^{\prime \prime }=s_{m_{k}}^{\prime }/j_{k}$. The zero-mean normalization vector $U_{i}^{\prime }=\left(S_{m_{i1}}^{\prime },S_{m_{i2}}^{\prime },\cdots ,S_{m_{k}}^{\prime },\cdots ,S_{m_{iQ}}^{\prime }\right)$ of the VTCL consists of the following normalization constants:
  (12) $$S_{ij}^{\prime }=\int_{-\infty }^{\infty }\left|s_{ij}^{\prime \prime }\left(t\right)\right|dt$$
4. In the same way, the acquired impact signal $x=(x_{1},x_{2},\cdots ,x_{Q})$ to be located is decomposed by EMD and the zero-mean normalized feature vector $z^{\prime }=\left(z_{1}^{\prime },z_{1}^{\prime },\cdots ,z_{Q}^{\prime }\right)$ is obtained, and the zero-mean normalization constant is:
  (13) $$Z_{j}=\int_{-\infty }^{\infty }\left|z_{j}^{\prime }\left(t\right)\right|dt$$
5. Carry out the ZNCC operation between the feature vectors of sample impact signal and the to-be-located impact signal. The VTCL comprehensive cross-correlation value between x and the impact sample signal $M_{i}$ is calculated as:
  (14) $$\gamma_{i}=\sum_{j=1}^{N}\left(\frac{s_{ij}^{\prime \prime }}{S_{ij}^{\prime }}*\frac{z_{j}^{\prime }}{Z_{j}}\right)\left(\tau \right)$$
6. Compare numerical values of $\gamma_{1}$, $\gamma_{2}$⋯$\gamma_{C}$, and take the points $\gamma_{max1}$, $\gamma_{max2}$, $\gamma_{max3}$,$\gamma_{max4}$ with the first four largest values to determine impact localization reference area. Define the point where the cross-correlation value of the $\gamma_{max1}$, $\gamma_{max2}$ connection interpolation is 1 as $c_{1}$, the point where the cross-correlation value of the $\gamma_{max1}$, $\gamma_{max3}$ connection interpolation is 1 as $c_{2}$, the point where the cross-correlation value of the $\gamma_{max1}$, $\gamma_{max4}$ connection interpolation is 1 as $c_{3}$, take $c_{1}$, $c_{2}$, $c_{3}$ as the boundary points of the impact localization reference area. Calculate the centroid of the reference area as the identified impact location.

## 3. Impact Experiment and Result Analysis

### 3.1. Impact Experimental System of the Variable Thickness Composite Laminate

**Figure 1:**
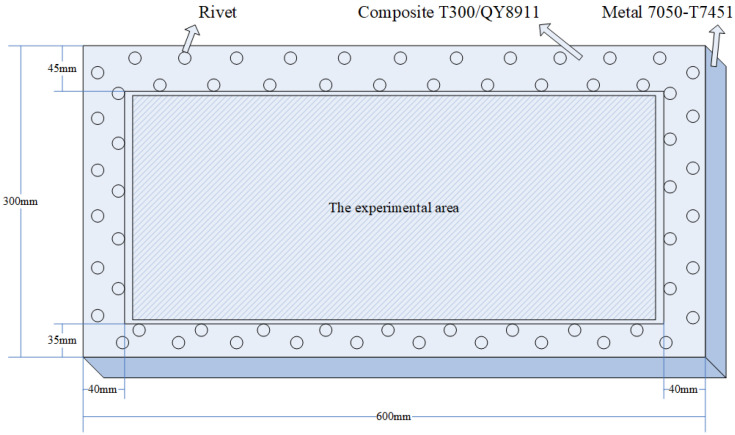
The composite laminate on the surface of the wing tank specimen and the impact experimental area.

**Figure 2:**
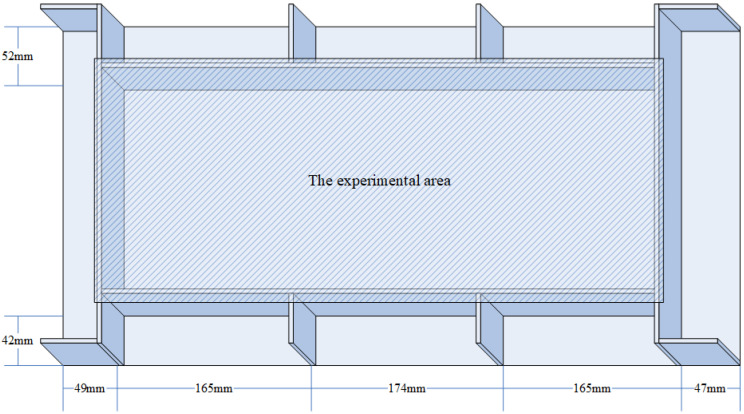
The fixation of the composite laminate on the tank surface.

**Figure 3:**
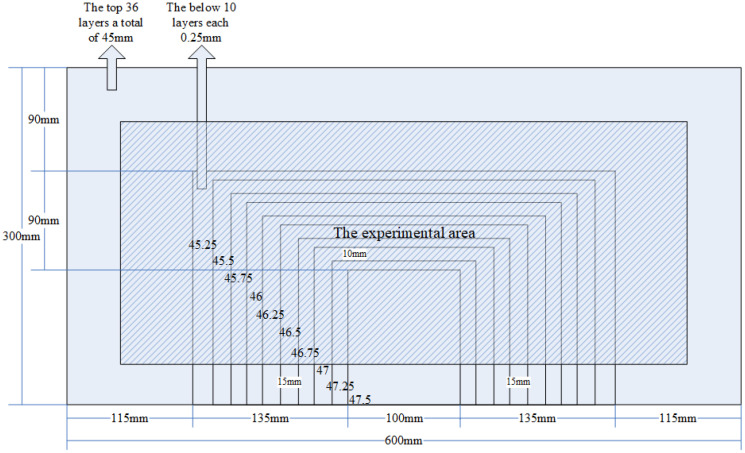
Change of ply thickness of composite laminate on the tank surface.

**Figure 4:**
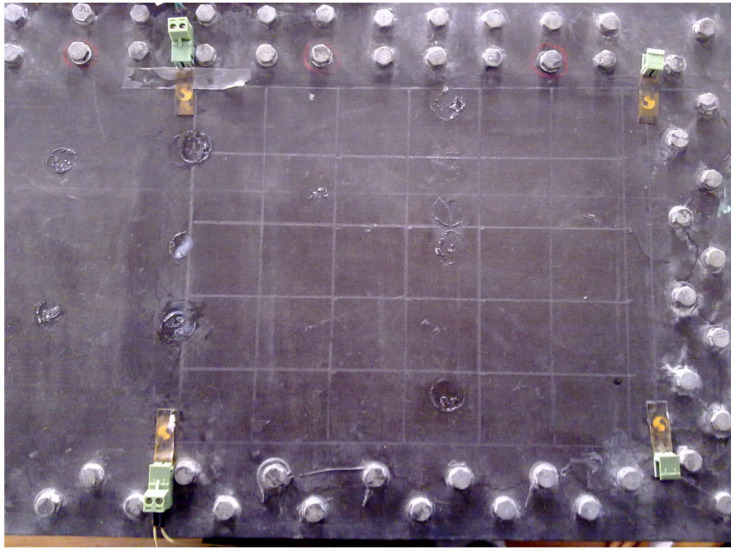
The impact experimental area of the composite laminate on the tank surface.

**Figure 5:**
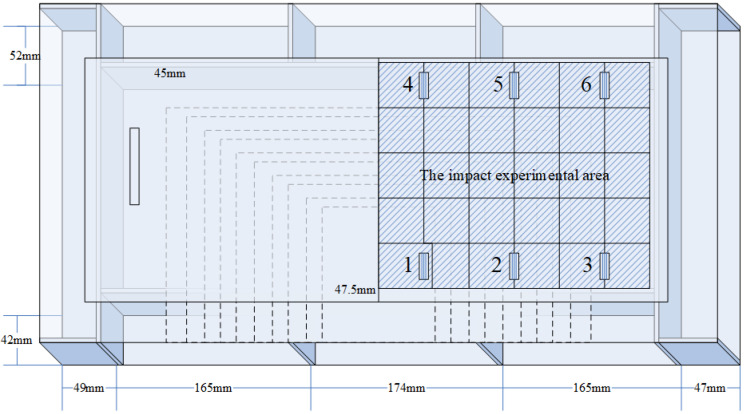
The variable thickness composite laminate on the tank surface.

The specimen used in the experiment is a composite wing tank as shown in Figure 1, and the size is 600 mm × 300 mm × 300 mm. The specimen is fixed on four sides, in which the frame width of the metal fixed support is 40 mm. The VTCL on the surface of specimen and its impact experimental area is shown in Figure 1 and uses rigid support. As shown in Figure 2, the composite material is T300/QY8911. The changes of the ply thickness of the composite laminate are shown in Figure 3. The thinnest part of the laminate is 45 mm, and the thickest part is 47.5 mm. The effective area of this four-sides fixed composite laminate is 520 mm × 220 mm. The half area of 240 mm × 200 mm is divided into 5 rows, 6 columns, 40 mm by 40 mm grids for the experiment. The experimental area is basically a variable thickness area, as shown in Figure 4 and Figure 5.

In the study of the impact of VTCL, the sensor arrangement and impact point selection should be noted: the impact point can completely and correctly excite each natural frequency below 125 Hz of the VTCL; the impact signal should reflect the structural impact characteristics correctly and be convenient for facilitating post-test data analysis; the sensor should not be arranged around the vulnerable area of impact to avoid damage.

**Figure 6:**
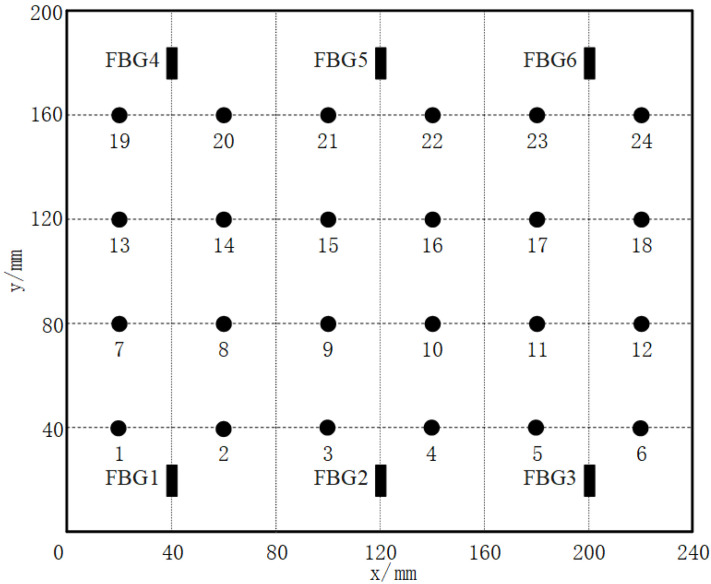
The sensor arrangement and impact points.

Therefore, based on the results of the vibration test, symmetry, fixation, and mechanical analysis of the VTCL on the surface of the wing tank, six FBG sensors were attached to the VTCL. The grating length is 10 mm. According to the characteristics of the composite specimen with variable thickness, the arrangement of the sensor is shown in Figure 5 and Figure 6, and the wavelength and position of each sensor are shown in Table 1.

**Figure 7:**
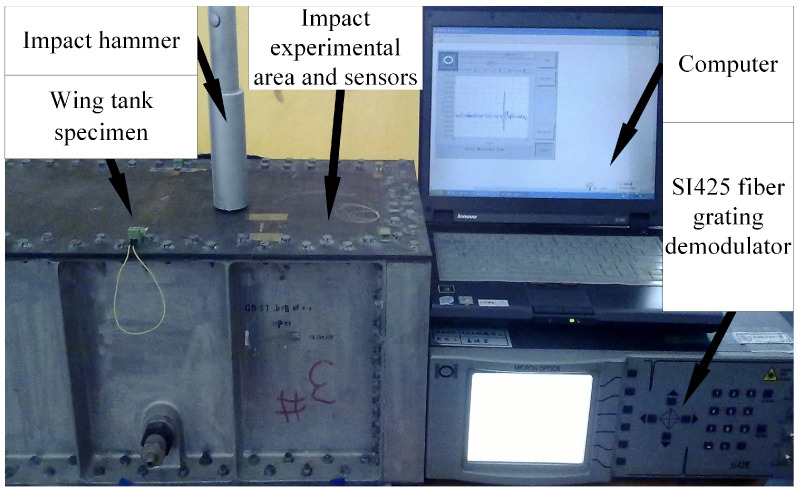
Impact monitoring system of a wing tank based on FBG sensors.

The impact monitoring system is shown in Figure 7, and the hammer impact method was adopted in the impact experiment. The impact monitoring system consists of impact hammer, specimen, FBG sensors, SI425 FBG demodulator (frequency 250 Hz, resolution 1 pm), and a computer. Six FBG sensors were divided into three lines connected to the demodulator which transmits the impact signal to the computer.

### 3.2. Establishment of Impact Sample Signal Database and Impact Signal Analysis

The impact sample signal database of the VTCL was established by the impact feature vectors. In order to establish the database, we used an impact hammer to impact the midpoint of 24 grid lines in the impact experimental area, as shown in Figure 6. The impact signal of each sample point was continuously collected at 250 Hz sampling frequency for 0.5 s. The impact energy of the hammer was set to 1 J.

In the following, the feature extraction method of FBG impact signals on VTCL is analyzed.

**Figure 8:**
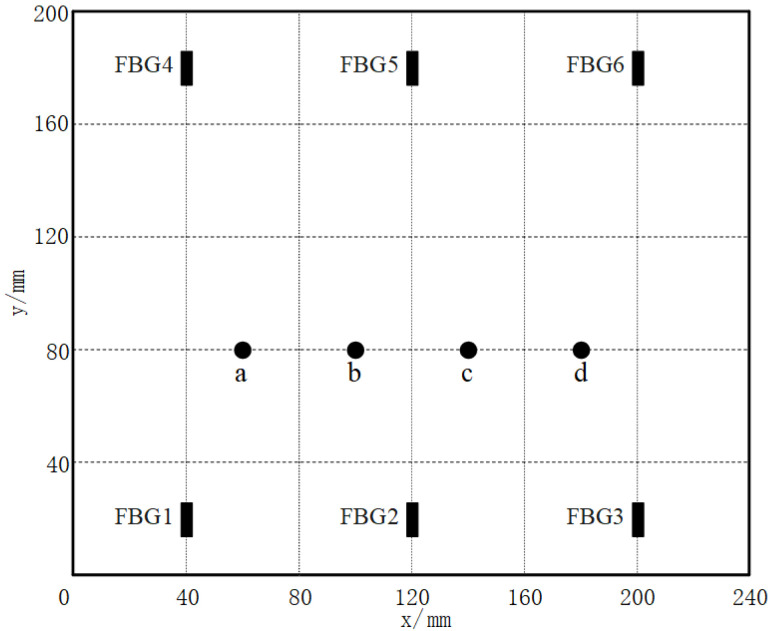
The impact points a–d.

**Figure 9:**
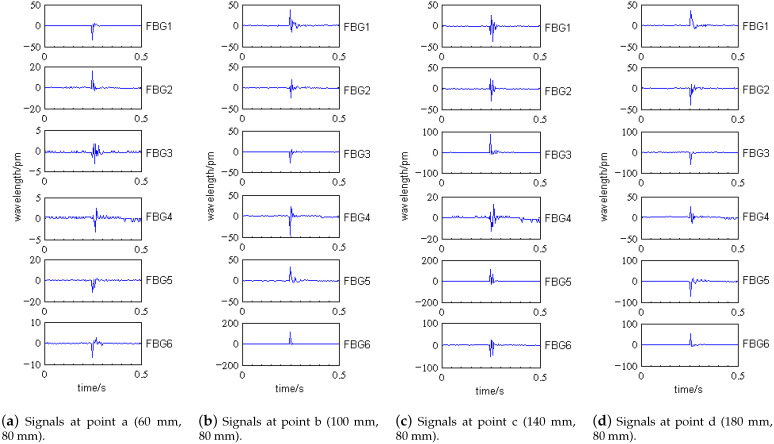
Signals at impact points a–d under 1 J.

**Figure 10:**
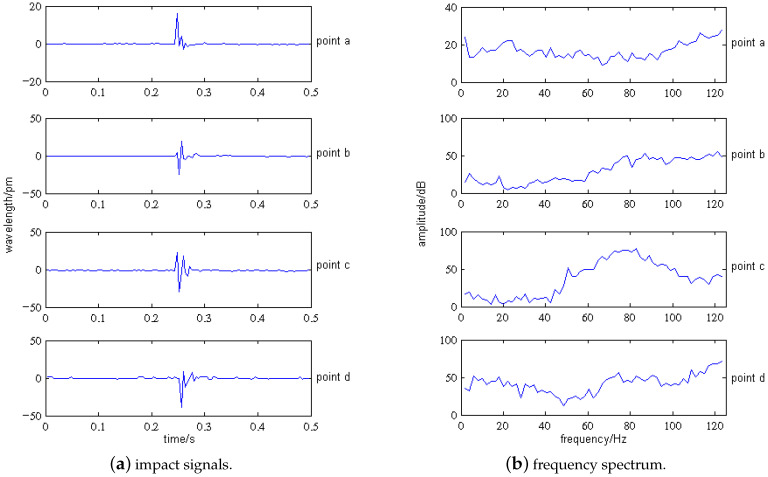
Signals and spectrogram of sensor No. 2 under 1 J.

Taking four impact points, a (60 mm, 80 mm), b (100 mm, 80 mm), c (140 mm, 80 mm), and d (180 mm, 80 mm) on the tank specimen as an example, sensor No. 2 is selected to analyze the impact signals. Figure 8 shows the locations of the impact points on the specimen. The impact signals collected by SI425 FBG demodulator are shown in Figure 9, in which the four-points impact signals detected by the sensor No. 2 are shown in Figure 10a.

As shown in Figure 10a, for the different impact points, the center wavelength shift of sensor No. 2 varies from 16 pm to 39 pm. We can get the following conclusions by analysis:

1. When impact points are at the same distance from the sensor and the thickness of the composite laminate at the impact positions are the same, the influence of impact position on signal cannot be judged by time-domain analysis. For example, the distances from the sensor No. 2 to the impact point b and c are equal, the composite laminate thickness at point b and c is basically the same, then the amplitude (FBG center wavelength shift of sensor No. 2) difference of impact signals at point b and c is relatively small.
2. When impact points are at the same distance from the sensor, but the thicknesses of the composite laminate at the impact positions are different, the time-domain analysis can be used to judge the influence of impact positions. The smaller the thickness, the greater the signal amplitude. For example, the distances from the sensor No. 2 to the impact point a and d are equal, but the composite laminate thickness at two points varies greatly; then, the amplitude of impact signals at two points differs greatly.

The impact time domain signals of other sensors in Figure 9 show that the center wavelength shift ranges from 5 pm to 125 pm, and the vibration duration of the signal is no more than 0.1 s. We can get a conclusion similar to that in Figure 10a: it is impossible to extract effective impact location features by using time-domain signals.

Fourier transform is performed on the time domain impact signal of the specimen collected by FBG sensors to obtain the corresponding spectrum, as shown in Figure 10b, which shows that there is no obvious resonant frequency for the VTCL. This is related to the variable section characteristics of the tank specimen. Each layer of composite material corresponds to a particular resonant frequency. When the thickness is constant, the entire laminate has one or two fixed resonant frequencies. However, when the thickness varies stepwise, the entire laminate has multiple fixed resonant frequencies. As the impact location changes, the amplitude of each resonant frequency changes. In the whole frequency band (5 Hz–125 Hz), the mean value of spectrum amplitude is consistent with the variation trend of wavelength amplitude at the same impact point. The mean value of the spectrum amplitude is related to the thickness of each impact point. The amplitudes of impact point c and d are the largest, that of point b is medium, and that of point a is the smallest. In summary, the classical spectrum analysis cannot provide enough effective information for the impact location of VTCL; hence, we added EMD to analyze the impact signals of the VTCL specimen.

**Figure 11:**
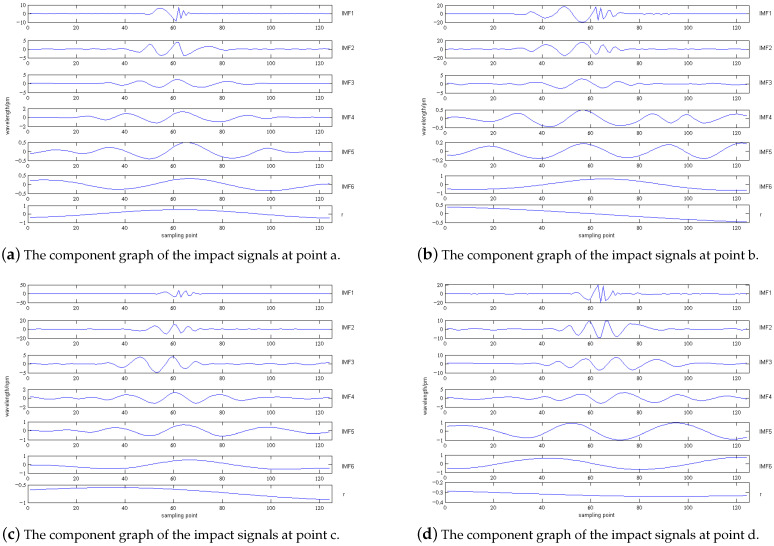
The EMD component graph of the impact signals at points a–d.

As shown in Figure 10a, the impact signals are decomposed by EMD to obtain the components of each order. After decomposition, six components and one residual are obtained. Figure 11 shows the six components and one residual obtained after the initial signal decomposition, labeled as IMF1–IMF6 and *r*.

**Figure 12:**
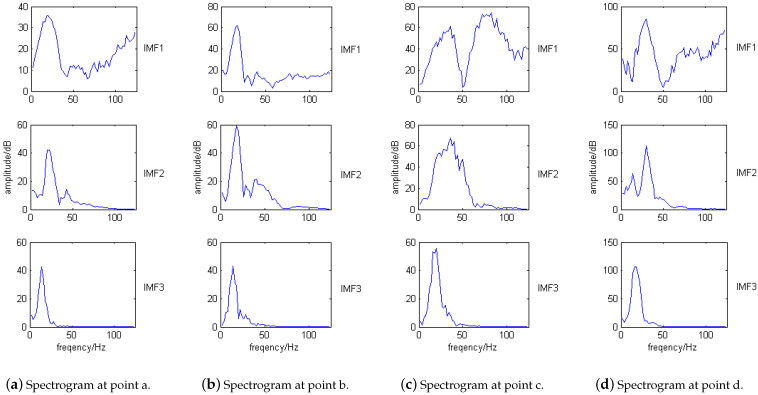
Spectrum of the first three-order EMD components at points a–d.

The IMF components are taken as the variable $x_{1}$ in Equation (4), the initial impact signal shown in Figure 10a is taken as the variable $x_{2}$. Table 2 is the correlation table for each IMF component and the initial signal. From Table 2, it can be concluded that the correlation coefficients of the first three orders are relatively larger with a maximum value of 0.3283 and a minimum value of 0.1749. Since the fourth-order, the correlation coefficients become very small, less than 0.1. Perform spectrum analysis on the first three order decomposition components, and the results are shown in Figure 12.

Figure 12 shows the spectrums of the first three-order EMD components of the impact signals. The spectrums at points a–d show that the impact natural frequencies of the VTCL are about 15 Hz, 20 Hz, 30 Hz, 40 Hz, 70 Hz, 80 Hz, 90 Hz, 110 Hz. The natural frequencies are estimated by the frequencies corresponding to multiple peaks in each order spectrum diagram. We can get the following conclusions:

1. The frequency band range of the EMD components gradually decrease to the low frequency direction from the first-order to the third-order. The second-order EMD component seldom detects impact signals above 40 Hz because its frequency band decreases to less than 40 Hz, and the third-order component can no longer detect the impact signal above 20 Hz because its frequency band is reduced to within 20 Hz;
2. The 20 Hz and 40 Hz spectral amplitudes of the first-order EMD component are lower than the second-order EMD component, and the 20 Hz spectral amplitude of the second-order EMD component is lower than the third-order;
3. With the change of impact position, the amplitude of resonance frequency of each EMD component changes.

For example, when the laminate thickness at point c is less than that of point a, the first order EMD component of point c excites less spectral amplitude, which is related to the layer thickness. Comparing Figure 11 and Figure 12, it can be concluded that the impact signals after EMD can distinguish the higher frequency signals from the lower, and the high frequency signals can also be more pronouncedly manifested. This provides a good basis for the impact monitoring research of VTCL.

**Figure 13:**
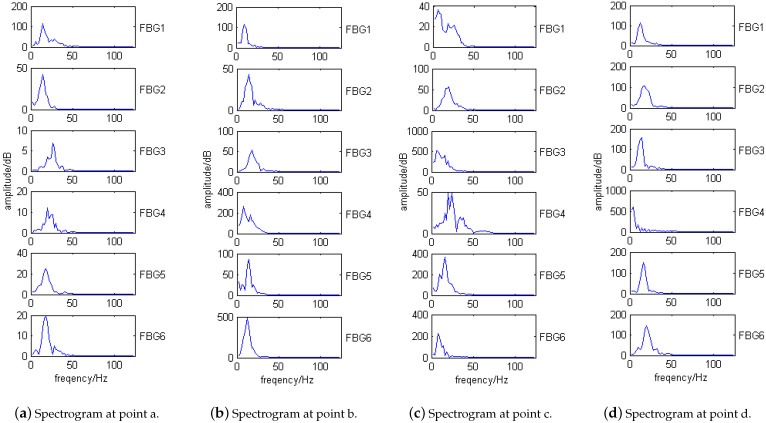
The third-order spectrum of the EMD.

The EMD algorithm is used to decompose the impact signals of each sensor, and the third order component is taken as an example for spectrum analysis, as shown in Figure 13. The frequency of third-order component is below 40 Hz, and we can get the following conclusions:

1. The spectrum amplitude of the third order component at each impact point is closely related to the distance between sensor and the impact point, and the laminate thickness at the sensor’s location.
2. The frequency band range of the third order component at each impact point is related to the positions of sensor and impact point.

Due to the anisotropic properties of the composite material, the physical properties of the material also behave differently in different directions. The natural frequencies of the same order stimulated by impacting tank specimen at different locations are also deviated. For VTCL, the natural frequencies of signals at different impact positions are different. Therefore, based on the spectrum of the third-order EMD component, preliminary impact localization studies can be conducted. However, according to the structural characteristics of VTCL, we need to reduce the influence of thickness factor on the accuracy of positioning results through a thickness coefficient, so as to establish a more accurate impact signal database for the variable thickness structure.

Based on the above analysis, a preliminary analysis of impact signals of tank specimen was carried out. The natural frequencies of VTCL and the characteristic information of the FBG sensor network were studied, which provides a basis for the impact monitoring and positioning research of VTCL.

### 3.3. Impact Localization Analysis

In order to verify the performance of the proposed localization method, 10 impact points were selected on the VTCL specimen for the verification experiment, and the influence of impact signal normalization and thickness correction on localization performance was studied. The performance of this method is evaluated by a localization error which is calculated from the absolute distance between the actual impact location and the estimated impact location.

**Figure 14:**
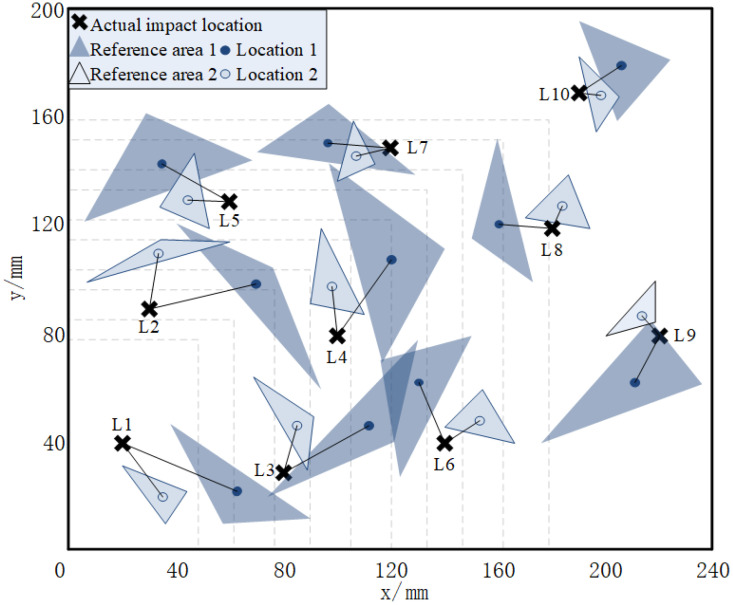
Verification experimental results of impact localization.

Figure 14 shows the impact localization results of 10 verification points. Area No. 1 is the localization reference area based on the normal cross-correlation localization (CL) method. Area No. 2 is the localization reference area based on the variable-thickness normalization CL method with thickness correction. Position No. 1 is the impact localization result based on the normal CL method, and position No. 2 is based on the variable-thickness normalization CL method—wherein, when using the method of normal CL, the localization error of verification point No. 1 and point No. 2 is larger than one-grid size (40 mm). Figure 14 shows the comparison between the two methods, in which the variable-thickness normalized CL method is obviously better than the normal CL method.

As the normalization step can reduce the effect of amplitude changes of impact signals on the localization accuracy, and relatively centralize the localization reference area, it can reduce the localization error effectively—especially when the impact point is located in the boundary area or larger thickness area, which can decrease the impact signal amplitude greatly, and leads to the error increase. The normalization step has a significant improvement for the impact localization results in the above cases. At the same time, the change of the laminate thickness at the impact point affects the detection sensitivity, while the thickness correction step can increase the localization performance under the above situation. As shown in Figure 14 and Table 3, the impact localization was performed by using a variable thickness normalized cross-correlation algorithm, the average error of 10 verification points is sharply reduced from 29.47 mm to 15.67 mm, and the maximum error changed from 45.31 mm to 24.41 mm. From the results, it can be concluded that the steps of variable thickness normalization do affect the maximum error value and the average error range of the localization results.

Furthermore, if we have a considerable number of impact sample signals, and the signals are measured in a series of FBG sensors, the effectiveness of this method is discussed as follows:

1. If the difference between the impact occurrence time of the two sample signals is greater than about 0.2 s, according to the conclusion obtained from Figure 9 (“the vibration duration of the signal is no more than 0.1 s”), it can be inferred that their time domain signals do not overlap, that is, the two signals do not affect each other. Then, we can use the proposed method to analyze and locate the two signals respectively.
2. If two signals affect each other, this method is invalid unless each single signal can be extracted without loss.
3. If the number of impact signals exceeds two, the proposed localization method can also be used for analysis in the same way.

In summary, the ZNCC localization algorithm can successfully locate the impact based on FBG sensors, and thickness correction step can improve the localization accuracy effectively. Experimental results show that, for the VTCL wing tank specimen, the maximum positioning error is 24.41 mm, and the average positioning error is 15.67 mm. The positioning accuracy is in line with the engineering application scope, in which the average error of sensor positioning is significantly reduced from 29.47 mm to 15.67 mm after adding variable thickness normalization procedure.

## 4. Conclusions

The various LVI loads on the composite laminates in service may cause the internal peeling, fracture, and delamination, which is difficult to detect from the outside. Therefore, the study on LVI of composite laminates can provide the basis for the damage mechanism and tolerance design of composite materials, and is very important for strengthening the application of composite design.

To locate the impact signal of VTCL on the surface of wing tank collected by FBG sensors, a localization method based on EMD, ZNCC, and thickness correction was proposed. This method does not need prior knowledge, and can effectively eliminate cross-interference of temperature on the sensor center wavelength, and is robust to impact energy. The EMD algorithm can effectively extract the useful feature information from the impact signals, while the ZNCC algorithm can effectively remove the temperature cross-sensitivity and impact energy influencing factors of the sensor, and the thickness correction can reduce the interference of variable thickness characteristics on localization performance.

1. We proposed a method for monitoring the LVI location of VTCL by the FBG sensing network. The results show that the FBG sensor can be used to study the impact of VTCL.
2. Based on the strain characteristics of FBG and the impact signal characteristics of composite structures, an impact localization method based on EMD, ZNCC, and thickness correction for VTCL is proposed. This method does not need prior knowledge, and can effectively eliminate cross-interference of temperature on the sensor center wavelength, and is robust to impact energy. The EMD method can more accurately extract the impact signal feature of VTCL. High frequency signal and low frequency signal can be distinguished by EMD decomposition, which provides a good basis for the impact study of VTCL. By using ZNCC and thickness correction, the localization can be achieved by comparing the impact feature. In addition, the complete impact localization procedures of the VTCL are given.
3. Based on the impact localization method of the variable thickness structures, an impact localization system for the VTCL is built. The system addresses impact monitoring of complex composite structures and is less susceptible to environmental disturbances. The performance of the proposed impact localization method is verified by experiments, which show that the method can accurately evaluate the impact location under the same conditions. The maximum positioning error is 24.41 mm and the average positioning error is 15.67 mm, which can meet the engineering application requirements of LVI load location discrimination. The variable-thickness normalization procedure (especially when the impact amplitude is smaller) significantly improves the localization performance, with a significant reduction in localization error from 29.47 mm to 15.67 mm.

## Figures and Tables

**Table 1:** Wavelength and position of the gratings.

Sensor	Center Wavelength/nm	Location/mm
FBG1	1529.939	(40, 20)
FBG2	1527.045	(120, 20)
FBG3	1535.118	(200, 20)
FBG4	1530.090	(40, 180)
FBG5	1555.793	(120, 180)
FBG6	1535.064	(200, 180)

**Table 2:** Correlation table for each order IMF of the EMD algorithm.

IMF1	IMF2	IMF3	IMF4	IMF5	IMF6	*r*
0.3283	0.2387	0.1802	0.0718	0.0661	0.0567	0.0226
0.3267	0.2442	0.1797	0.0823	0.0698	0.0606	0.0297
0.3181	0.2378	0.1846	0.0884	0.0763	0.0673	0.0289
0.3085	0.2281	0.1749	0.0786	0.0664	0.0574	0.0261

**Table 3:** The localization results of 10 verification points.

Impact Point (mm)	Normal Cross-Correlation Localization Results (mm)	Normal Cross-Correlation Localization Error (mm)	Variable-Thickness Normalized Cross-Correlation Localization Results (mm)	Variable-Thickness Normalized Cross-Correlation Localization Error (mm)
1 (20, 40)	(62, 23)	45.31	(34, 20)	24.41
2 (30, 90)	(69, 99)	40.03	(32, 111)	21.10
3 (80, 30)	(112, 47)	36.24	(85, 48)	18.68
4 (100, 80)	(120, 109)	35.23	(98, 99)	19.11
5 (60, 130)	(34, 143)	29.07	(43, 131)	17.03
6 (140, 40)	(130, 63)	25.08	(152, 49)	15
7 (120, 150)	(96, 152)	24.08	(107, 148)	13.15
8 (180, 120)	(160, 122)	20.10	(183, 129)	9.49
9 (220, 80)	(211, 62)	20.12	(213, 88)	10.63
10 (190, 170)	(206, 181)	19.42	(198, 169)	8.06

## Data Availability

The data presented in this study are available on request from the corresponding author.
